# A Pilot Study on the Effects of Slow Paced Breathing on Current Food Craving

**DOI:** 10.1007/s10484-017-9351-7

**Published:** 2017-02-14

**Authors:** Adrian Meule, Andrea Kübler

**Affiliations:** 1 Department of Psychology, University of Salzburg, Hellbrunner Straße 34, 5020 Salzburg, Austria; 2 Center for Cognitive Neuroscience, University of Salzburg, Hellbrunner Straße 34, 5020 Salzburg, Austria; 3 Institute of Psychology, University of Würzburg, Würzburg, Germany

**Keywords:** Food cues, Food craving, Eating behavior, Slow paced breathing, Heart rate variability, Biofeedback

## Abstract

Heart rate variability biofeedback (HRV-BF) involves slow paced breathing (approximately six breaths per minute), thereby maximizing low-frequent heart rate oscillations and baroreflex gain. Mounting evidence suggests that HRV-BF promotes symptom reductions in a variety of physical and mental disorders. It may also positively affect eating behavior by reducing food cravings. The aim of the current study was to investigate if slow paced breathing can be useful for attenuating momentary food craving. Female students performed paced breathing either at six breaths per minute (*n* = 32) or at nine breaths per minute (*n* = 33) while watching their favorite food on the computer screen. Current food craving decreased during a first resting period, increased during paced breathing, and decreased during a second resting period in both conditions. Although current hunger increased in both conditions during paced breathing as well, it remained elevated after the second resting period in the nine breaths condition only. Thus, breathing rate did not influence specific food craving, but slow paced breathing appeared to have a delayed influence on state hunger. Future avenues are suggested for the study of HRV-BF in the context of eating behavior.

## Introduction

Craving refers to an intense desire to consume a substance such as alcohol, tobacco, or a specific food (Hormes and Rozin 2010). In Western societies, the most often craved food is chocolate (Rozin et al. 1991; Weingarten and Elston 1991). Food craving is a common experience, that is, it is experienced by most people from time to time. There is, however, a sex difference such that women more frequently experience food craving than men (Cepeda-Benito et al. 2003; Weingarten and Elston 1991). Furthermore, although experiencing food craving is normal, more frequent and intense food cravings are associated with low dieting success, overweight, and binge eating (Franken and Muris 2005; Gendall et al. 1997; Meule et al. 2017; Potenza and Grilo 2014).

Because of its relevance in eating and weight regulation, a growing body of research has examined different techniques that may reduce food craving, for example bibliotherapy (Rodríguez-Martín et al. 2013), transcranial brain stimulation (Grall-Bronnec and Sauvaget 2014; Jansen et al. 2013), brief guided imagery and body scanning (Hamilton et al. 2013) or acceptance-based and other mindfulness strategies (Alberts et al. 2010; Lacaille et al. 2014). Another strategy for controlling food craving may be slow breathing such as during heart rate variability biofeedback (HRV-BF; Meule et al. 2012a).

Heart rate variability refers to the variation of intervals between two heartbeats over a certain amount of time (e.g., several minutes). Accordingly, the most basic index of HRV is the standard deviation of heartbeat intervals (standard deviation of normal-to-normal beats, SDNN). By spectral analysis, the heartbeat time series can also be partitioned into different oscillatory components, two of which are relevant for the current investigation: a low frequency (LF) band (0.04–0.15 Hz), which represents oscillations related to baroreflex activity, and a high frequency (HF) band (0.15–0.4 Hz), which reflects effects of respiration on heart rate (Reyes del Paso et al. 2013). High HRV represents a well-functioning cardiovascular system that can flexibly adapt to changing situational demands and, thus, is associated with physical health (Thayer et al. 2010). In addition, as HRV is under vagal control (i.e., the tenth cranial nerve), it has been found that it is associated with activity in prefrontal and limbic brain areas and, consequently, with mental health (Appelhans and Luecken 2006; Thayer et al. 2012; Thayer and Lane 2009). For example, higher resting HRV has been related to higher self-regulation, functional emotion regulation, and higher well-being (Geisler et al. 2010; Meule et al. 2013; Reynard et al. 2011; Segerstrom and Nes 2007).

It has also been found that HRV is associated with substance craving and eating-related self-regulation. In a recent study in alcohol-dependent patients, for example, HRV was inversely related to self-reported alcohol craving (Quintana et al. 2013). With regard to eating behavior, it has been found that lower HRV was related to lower dieting success in restrained eaters (Meule et al. 2012c, d) and to higher disordered eating in chocolate cravers (Rodríguez-Ruiz et al. 2009).

Only few studies have examined if interventions that modulate HRV such as HRV-BF can be applied as a craving regulation strategy. Heart rate variability biofeedback involves slow paced breathing (usually at 5.5–6.0 breaths per minute or 0.1 Hz), which stimulates baroreflex activity and increases HRV, particularly in the LF power band (Brown et al. 1993; Lehrer 2013). In one study with individuals exhibiting post traumatic stress disorder symptoms, there was a trend toward a decrease of drug craving after several weeks of practicing HRV-BF (Zucker et al. 2009). Most recently, no craving reducing effect of HRV-BF could be observed in substance use disorder inpatients, but it appeared that HRV-BF dissociated the relationship between baseline HRV and changes in craving (Eddie et al. 2014). In another study, participants who scored high on trait food craving practiced HRV-BF for 4 weeks (12 sessions with a duration of 20 min each) and reported a decrease in food craving experiences and higher control over their cravings after the intervention (Meule et al. 2012a). To conclude, preliminary evidence suggests that practicing HRV-BF may aid in reducing craving for food or other substances.

The mediating mechanisms of these effects, however, are unknown. They may relate to stimulation of vagal afferent pathways that affect brain areas involved in affect regulation, craving regulation and self-control and/or to stimulation of vagal efferent pathways related to relaxation (Lehrer and Gevirtz 2014). Yet, another possibility is that participants incorporate the breathing technique they acquired during HRV-BF into their daily routine and use it as an immediate craving regulation strategy whenever a craving emerges. However, this possibility has not been tested yet and little is known about the immediate effects of slow paced breathing or HRV-BF on craving in general and on food craving in particular.

Some studies in smokers suggest that slow breathing momentarily attenuates the desire to smoke (McClernon et al. 2004; Shahab et al. 2013). Interestingly, although HRV was not assessed in the study by McClernon and colleagues (2004), they instructed participants to inhale for 5 s and exhale for 5 s, which exactly matches the breathing rate usually applied during HRV-BF. Overall, self-reported appetite did not change as a function of slow breathing, but it was found that appetite decreased in those who reported more years of smoking, smoking for relaxation and smoking for habit withdrawal (McClernon et al. 2004). In another study, in which food-related thoughts were used to induce food craving, an instruction to focus on breathing did not attenuate food craving (May et al. 2010). The authors speculated that directing participants’ attention to their abdomen may have drawn attention to any physiological hunger sensations, thereby hampering a possible craving reducing effect of breath focus. To conclude, research on immediate effects of slow paced breathing on craving is scarce and to date, no study has been conducted on immediate effects of slow paced breathing or HRV-BF on food craving.

In the current study, female students were investigated in a single session of paced breathing at six breaths per minute (experimental condition) or at nine breaths per minute (control condition). To induce food craving, a food picture was presented on the computer screen during paced breathing. As related studies yielded inconclusive findings, a non-directional hypothesis was formulated about how breathing rate would influence current food craving. It was hypothesized that state food craving would be differentially influenced by breathing rate: slow paced breathing at six breaths per minute could result in either a reduction of current food craving, similar to what has been found for tobacco craving (McClernon et al. 2004), or it could even facilitate current food craving, similar to what has been speculated during breath focus (May et al. 2010). As a manipulation check (i.e., to examine if participants adhered to the breathing instructions), HRV was analyzed. Specifically, time domain measures of HRV (e.g., SDNN) and LF power (but not HF power) markedly increase when breathing at six breaths per minute as compared to faster breathing rates (Brown et al. 1993; Tsai et al. 2015). Thus, it was expected that SDNN and LF power (but not HF power) would be higher during paced breathing in the six breaths condition than in the nine breaths condition. These differences were expected to be absent during a resting period *after* paced breathing, indicating that participants returned to their usual breathing rates. However, as has been found in previous studies (Wells et al. 2012), it was expected that participants in the six breaths condition would have higher HF power than those in the nine breaths condition *after* the paced breathing period, indicating higher cardiac vagal tone.

## Method

### Participants

Female students, who received course credits for participation, were recruited at the University of Würzburg (Würzburg, Germany). Only women were recruited to avoid a possible confounding influence of sex, as there are sex differences in food craving among other eating-related variables (Cepeda-Benito et al. 2003; Hormes 2014; Hormes et al. 2014). Exclusion criteria were the presence of cardiovascular or respiratory diseases and pregnancy. Sixty-six women participated in the study, but one participant was excluded from analyses because of a very low body mass index (BMI < 17.5 kg/m²), leaving a final sample size of *n* = 65. All remaining participants were normal-weight or overweight (*M* = 22.00 kg/m², *SD* = 2.71). Mean age was *M* = 21.20 years (*SD* = 2.91). Five women (7.7%) reported to be current smokers and 22 women (33.8%) reported to be current dieters.

### Materials

#### Food Cravings Questionnaire-State (FCQ-S).

The FCQ-S (Cepeda-Benito et al. 2000; Meule et al. 2012b) is a 15-item self-report measure for the assessment of the intensity of current food craving. This questionnaire was employed four times during the experiment and its scores constituted the main outcome measure. Items are scored on a 5-point scale ranging from *strongly disagree* to *strongly agree*. Its original versions contained five subscales representing (1) an intense desire to eat (e.g., “I’m craving one or more specific foods.”), (2) anticipation of positive reinforcement that may result from eating (e.g., “Eating one or more specific foods would feel wonderful.”), (3) anticipation of relief from negative states and feelings as a result of eating (e.g., “I would feel more alert if I could satisfy my craving.”), (4) lack of control over eating (e.g., “If I had one or more specific foods, I could not stop eating it.”), and (5) craving as a physiological state (e.g., “I am hungry.”)(Cepeda-Benito et al. 2000). In the validation study of its German version, it was found that the *desire to eat* and *lack of control* subscales as well as the *reinforcement* and *relief* subscales can be combined, resulting in three subscales (Meule et al. 2012b), which were also used in the current study. Internal consistencies across the four measurements ranged between Cronbach’s α = 0.70–0.86 for the desire to eat/lack of control subscale, α = 0.82–0.90 for the reinforcement/relief subscale, and α = 0.78–0.90 for the hunger subscale.

#### Food Cravings Questionnaire-Trait-reduced (FCQ-T-r)

The FCQ-T-r (Meule et al. 2014) is a 15-item short form of the FCQ-T (Cepeda-Benito et al. 2000; Meule et al. 2012b) for the assessment of the frequency and intensity of food craving experiences in general. This questionnaire was used to establish that conditions did not differ in trait food craving. Items are scored on a 6-point scale ranging from *never*/*not applicable* to *always*. Exemplary items are “Whenever I have cravings, I find myself making plans to eat.”, “Once I start eating, I have trouble stopping.”, or “It is hard for me to resist the temptation to eat appetizing foods that are in my reach”. Internal consistency was Cronbach’s α = 0.91 in the current study.

#### Eating Disorder Examination – Questionnaire (EDE-Q)

The EDE-Q (Fairburn and Beglin 1994; Hilbert et al. 2007) measures eating disorder psychopathology over the last 28 days. This questionnaire was used to establish that conditions did not differ in disordered eating behavior. It consists of 22 items and items are scored on a 7-point scale ranging from *no days*/*not at all* to *every day*/*markedly*. It comprises four subscales assessing (1) restraint (e.g., “Have you been deliberately trying to limit the amount of food you eat to influence your shape or weight [whether or not you have succeeded]?”, “Have you gone for long periods of time [8 waking hours or more] without eating anything at all in order to influence your shape or weight?”), (2) eating concern (e.g., “Has thinking about food, eating or calories made it very difficult to concentrate on things you are interested in [for example, working, following a conversation, or reading]?”, “Have you had a definite fear of losing control over eating?”), (3) weight concern (e.g., “Have you had a strong desire to lose weight?”, “Has your weight influenced how you think about [judge] yourself as a person?”), and (4) shape concern (e.g., “Have you had a definite desire to have a totally flat stomach?”, “Has your shape influenced how you think about [judge] yourself as a person?”). Only the total score was used in the current study and internal consistency was Cronbach’s α = 0.95.

#### Sociodemographic and Anthropometric Information

Participants reported their age (in years), if they were current smokers (yes/no), and the time since their last meal (in hours). Current dieting status (yes/no) was assessed with a single question (“Are you currently restricting your food intake to control your weight (e.g., by eating less or avoiding certain foods)?”; Meule et al. 2012c). Body height was measured with a body height meter and body weight was measured with a digital personal scale (PS 22, Beurer GmbH, Ulm, Germany) for computing BMI (kg/m²).

#### Heart Rate Recording

Heartbeat intervals were recorded with a chest strap and the polar watch RS800CX (Polar Electro Oy, Kempele, Finland; for a discussion of validity, see Quintana et al. 2012).

#### Paced Breathing

The stress pilot (Biocomfort Diagnostics GmbH & Co.KG, Wendlingen, Germany) was used for pacing breathing and presentation of the food stimuli. A pacing bar was shown for guiding breathing rate, which was set either at a breathing rate of six breaths per minute or at nine breaths per minute. The program allows for individual selection of a background picture and one food picture was selected individually for each participant (see below).

#### Stimuli

**Fig. 1:**
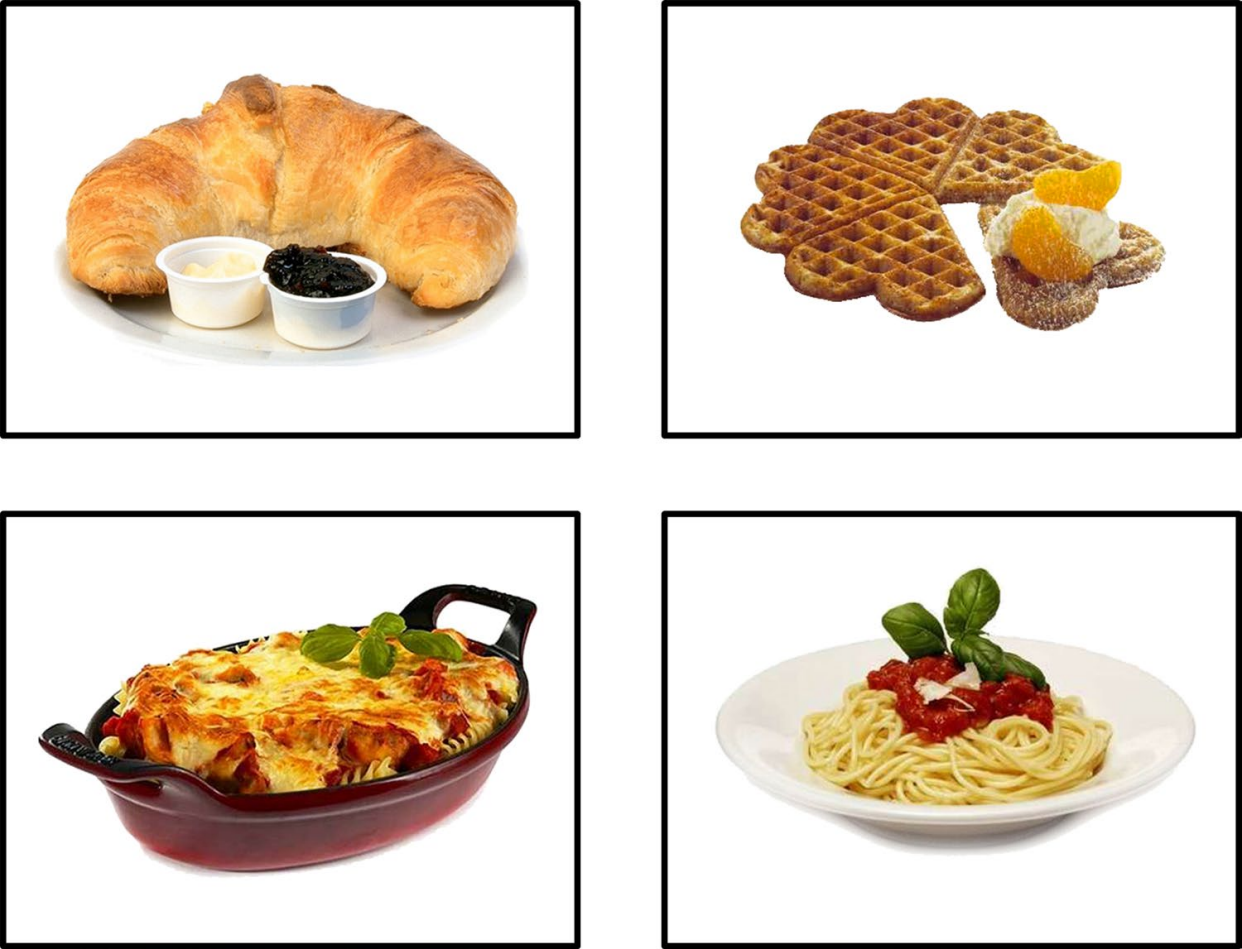
Exemplary food pictures from a set of 42 of which each participant could choose one. The four food pictures were chosen most often by the participants in the current study (Croissant: *n* = 10 [15.2%], waffle: *n* = 8 [12.1%], pasta bake: *n* = 7 [10.6%], spaghetti: *n* = 5 [7.6%])

Forty-two food pictures were selected from the *food.pics* database (Blechert et al. 2014).^1^ No specific criteria were used for this selection, except that the foods should be energy dense and that the picture set should contain a mixture of sweet and savory foods (cf. Fig. 1).

### Procedure

Participants were randomly assigned to one of the two experimental conditions and tested individually in the laboratory between 8 a.m. and 6:30 p.m. (*Mdn* = 12:15 p.m.). They were instructed to refrain from eating and consumption of caffeinated drinks at least 1 h before the experiment. Mean food deprivation (time since the last meal) was *M* = 5.26 (*SD* = 4.69) hours. After participants were welcomed by the experimenter, they read and signed informed consent. Then, they were shown the 42 food pictures on the computer screen and were asked to pick the one that they wanted to eat the most at the moment. Subsequently, participants were provided with the FCQ-S for the first time and were instructed to think of the food they had selected when completing the FCQ-S. Following this, heart rate was recorded for 10 min in supine position and participants were instructed to close their eyes and relax. After this resting period, participants completed the FCQ-S for the second time. Then, participants practiced paced breathing either at six or nine breaths per minute while their previously selected food picture was presented on the screen throughout for 10 min. Subsequently, they completed the FCQ-S for the third time and then heart rate was again recorded for 10 min at rest in supine position. Participants then completed the FCQ-S for the fourth time and completed the FCQ-T and EDE-Q, among others. Finally, body weight and height was measured.

### Data Analyses

Heart rate data were analyzed with the ARTiiFACT software (Kaufmann et al. 2011). Recordings were first screened visually, which revealed that, due to technical difficulties, psychophysiological data of some subjects (first resting period: *n* = 7, paced breathing: *n* = 18, second resting period *n* = 13) could not be used and were replaced by mean values for each condition separately^2^. Artifacts were corrected by cubic spline interpolation. As a general index of HRV, SDNN was computed. In the frequency domain, LF (0.04–0.15 Hz), and HF (0.15–0.4 Hz) power in ms² was determined by Fast Fourier Transformation.

To evaluate if randomization was successful, conditions were compared regarding age, BMI, food deprivation, trait food craving, and eating disorder symptomatology with independent *t*-tests. Regarding smoking and dieting status, conditions were compared with χ²-tests. To analyze changes in state food craving, separate analyses of variance for repeated measures were run with condition (six breaths vs. nine breaths) as between-subject factor, time (baseline vs. after first resting period vs. after paced breathing vs. after second resting period) as within-subject factor, and FCQ-S scores (desire to eat/lack of control, reinforcement/relief, hunger) as dependent variables.^3^ To analyze changes in HRV, separate analyses of variance for repeated measures were run with condition (six breaths vs. nine breaths) as between-subject factor, measurement (first resting period vs. paced breathing vs. second resting period) as within-subject factor, and HRV (SDNN, LF, HF) as dependent variables. Significant effects were followed up with *t*-tests. Exact *p*-values (two-tailed) are reported in case of significance (*p* ≤ 0.05), except when *p* < .001. *P*-values > 0.05 are displayed as *ns*.

## Results

### Sample Characteristics

Conditions did not differ in age, BMI, food deprivation, trait food craving, eating disorder symptomatology, or baseline food craving and hunger (Table 1). They also did not differ in smoking status (χ²_(1)_ = 0.25, *ns*) or dieting status (χ²_(1)_ = 0.38, *ns*).

**Table 1:** Sample characteristics

	Six breaths (*n* = 32) *M* (*SD*)	Nine breaths (*n* = 33) *M* (*SD*)	*t*	*p*
Age (years)	20.59 (2.01)	21.79 (3.50)	1.68	*ns*
Body mass index (kg/m²)	21.37 (2.10)	22.62 (3.10)	1.90	*ns*
Food deprivation (hours)	5.79 (5.14)	4.74 (4.21)	0.90	*ns*
Food Cravings Questionnaire-Trait-reduced	41.88 (10.40)	40.18 (10.17)	0.66	*ns*
Eating Disorder Examination-Questionnaire	1.51 (1.40)	1.20 (0.81)	1.09	*ns*
Food Cravings Questionnaire-State (baseline)				
Desire/control	17.03 (3.49)	18.12 (3.65)	1.23	*ns*
Reinforcement/relief	17.47 (4.27)	18.36 (4.87)	0.79	*ns*
Hunger	8.59 (3.14)	8.12 (2.68)	0.65	*ns*

### State Food Craving

#### Desire to Eat/Lack of Control

**Fig. 2:**
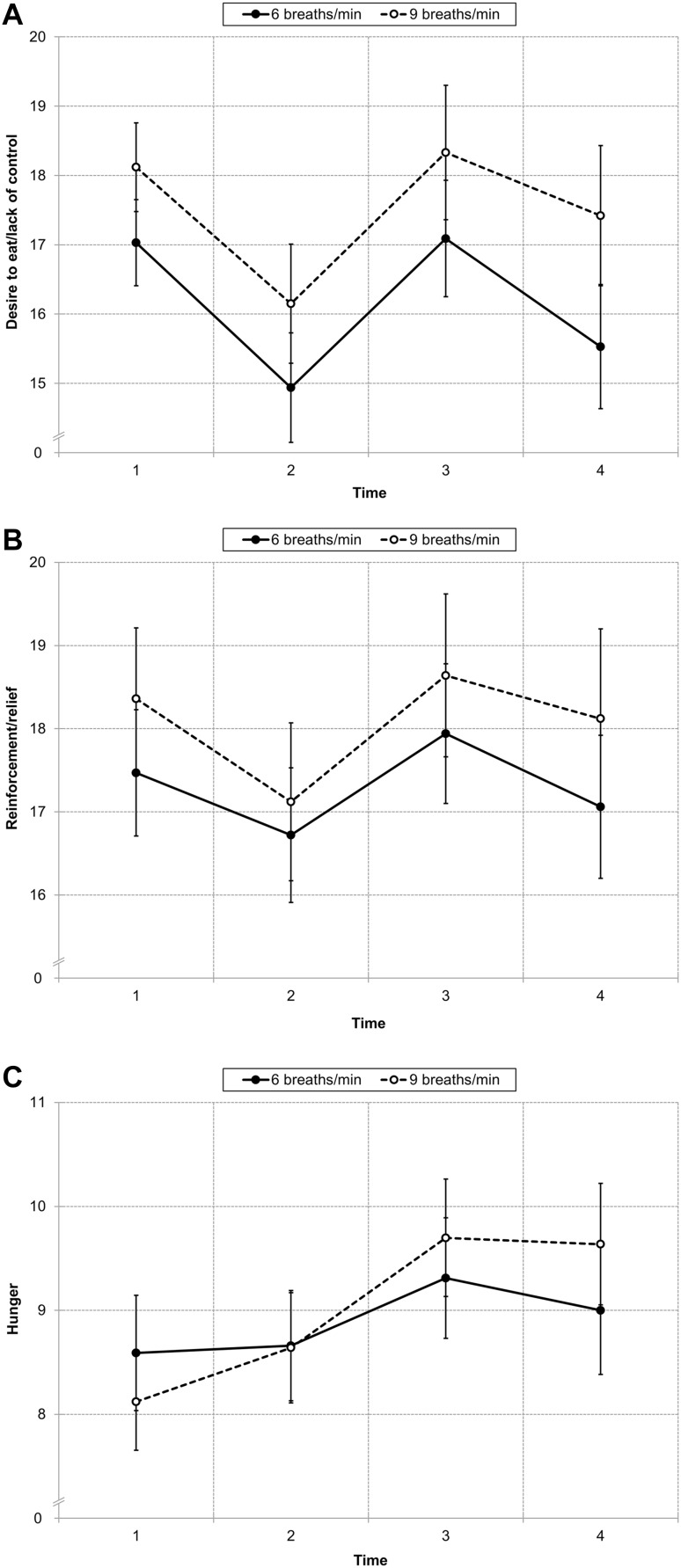
State food craving as a function of time and condition. In both conditions, desire to eat/lack of control (**a**) and reinforcement/relief (**b**) decreased during the first 10 min resting period (from time 1 to 2), increased during paced breathing (from time 2 to 3), and decreased during the second 10 min resting period (from time 3 to 4). Hunger (**c**) also increased during paced breathing (from time 2 to 3) in both conditions, but only in the nine breaths condition, hunger at the end of the experiment (time 4) was higher as compared to before and after the first resting period (time 1 and 2). *Error bars* indicate standard error of the mean

There was a main effect of time (*F*_(3,189)_ = 11.34, *p* < .001, η_p_² = 0.15), indicating that scores were decreased after the first resting period (*M* = 15.55, *SD* = 4.71) compared to before (*M* = 17.59, *SD* = 3.58, *t*_(64)_ = 5.43, *p* < .001), were increased after paced breathing (*M* = 17.72, *SD* = 5.20) compared to before (*t*_(64)_ = 5.33, *p* < .001) and were decreased again after the second resting period (*M* = 16.49, *SD* = 5.50, *t*_(64)_ = 3.34, *p* = .001; Fig. 2a). There was no main effect of condition (*F*_(1,63)_ = 1.62, *ns*) and no interaction condition × time (*F*_(3,189)_ = 0.36, *ns*).

#### Reinforcement/Relief

There was a main effect of time (*F*_(3,189)_ = 4.68, *p* = .004, η_p_² = 0.07), indicating that scores were decreased after the first resting period (*M* = 16.92, *SD* = 5.01) compared to before (*M* = 17.92, *SD* = 4.57, *t*_(64)_ = 2.91, *p* = .01), were increased after paced breathing (*M* = 18.29, *SD* = 5.20) compared to before (*t*_(64)_ = 4.29, *p* < .001) and were decreased again after the second resting period (*M* = 17.60, *SD* = 5.57, *t*_(64)_ = 2.11, *p* = .04; Fig. 2b). There was no main effect of condition (*F*_(1,63)_ = 0.42, *ns*) and no interaction condition × time (*F*_(3,189)_ = 0.28, *ns*).

#### Hunger

There was a main effect of time (*F*_(3,189)_ = 13.45, *p* < .001, η_p_² = 0.18), which was further qualified by an interaction of condition × time (*F*_(3,189)_ = 2.68, *p* = .05, η_p_² = 0.04). Scores were increased after paced breathing (six breaths: *M* = 9.31, *SD* = 3.29; nine breaths: *M* = 9.70, *SD* = 3.25) compared to both before (six breaths: *M* = 8.59, *SD* = 3.14, *t*_(31)_ = 2.26, *p* = .031; nine breaths: *M* = 8.12, *SD* = 2.68, *t*_(32)_ = 4.46, *p* < .001) and after (six breaths: *M* = 8.66, *SD* = 3.01, *t*_(31)_ = 3.07, *p* = .004; nine breaths: *M* = 8.64, *SD* = 3.03, *t*_(32)_ = 3.23, *p* = .003) the first resting period in both conditions (Fig. 2c). However, only in the nine breaths condition scores after the second resting period (*M* = 9.64, *SD* = 3.36) were still elevated compared to before (*t*_(32)_ = 4.49, *p* < .001) and after (*t*_(32)_ = 4.90, *p* < .001) the first resting period (Fig. 2c).^4^ There was no main effect of condition (*F*_(1,63)_ = 0.32, *ns*).

### Heart Rate Variability

#### SDNN

**Fig. 3:**
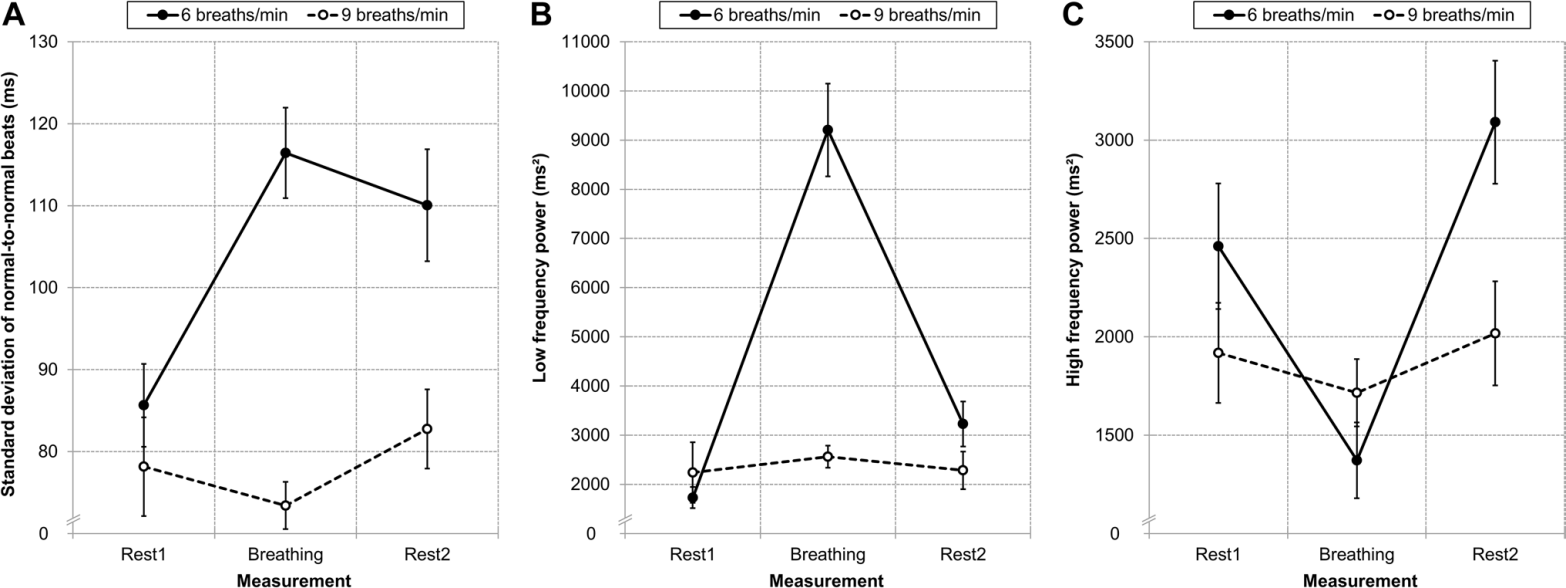
Heart rate variability as a function of measurement and condition. Conditions did not differ during the first 10 min resting period (Rest1). Participants in the six breaths condition had higher SDNN (**a**) and low frequency power (**b**) during paced breathing (Breathing) and higher SDNN (**a**) and high frequency power (**c**) during the second 10 min resting period (Rest2) than those in the nine breaths condition. *Error bars* indicate standard error of the mean

There were main effects of condition (*F*_(1,63)_ = 17.20, *p* < .001, η_p_² = 0.21) and measurement (*F*_(2,126)_ = 9.75, *p* < .001, η_p_² = 0.13), which were further qualified by an interaction of condition × measurement (*F*_(2,126)_ = 12.12, *p* < .001, η_p_² = 0.16). Participants in the six breaths condition had higher SDNN during paced breathing (*M* = 116.44, *SD* = 31.15) and the second resting period (*M* = 110.06, *SD* = 38.66) than those in the nine breaths condition (*M* = 73.42, *SD* = 16.52, *t*_(63)_ = 6.99, *p* < .001 and *M* = 82.77, *SD* = 27.76, *t*_(63)_ = 3.28, *p* = .002). Conditions did not differ during the first resting period (*t*_(63)_ = 0.95, *ns*; Fig. 3a).

#### LF Power

There were main effects of condition (*F*_(1,63)_ = 20.72, *p* < .001, η_p_² = 0.25) and measurement (*F*_(2,126)_ = 37.99, *p* < .001, η_p_² = 0.38), which were further qualified by an interaction of condition × measurement (*F*_(2,126)_ = 31.88, *p* < .001, η_p_² = 0.34). Participants in the six breaths condition had higher LF power during paced breathing (*M* = 9205.57, *SD* = 5346.87) than those in the nine breaths condition (*M* = 2560.96, *SD* = 1289.92, *t*_(63)_ = 6.94, *p* < .001). Conditions did not differ during the first (*t*_(63)_ = 0.78, *ns*) and second resting period (*t*_(63)_ = 1.59, *ns*; Fig. 3b).

#### HF Power

There was a main effect of measurement (*F*_(2,126)_ = 12.78, *p* < .001, η_p_² = 0.17), which was further qualified by an interaction of condition × measurement (*F*_(2,126)_ = 6.25, *p* = .003, η_p_² = 0.09). There was no main effect of condition (*F*_(1,63)_ = 2.30, *ns*). Participants in the six breaths condition had higher HF power during the second resting period (*M* = 3091.32, *SD* = 1768.89) than those in the nine breaths condition (*M* = 2017.38, *SD* = 1519.32, *t*_(63)_ = 2.63, *p* = .01). Conditions did not differ during the first resting period (*t*_(63)_ = 1.33, *ns*) and paced breathing (*t*_(63)_ = 1.33, *ns*; Fig. 3c).

## Discussion

In the current study, the immediate effects of slow paced breathing on current food craving were investigated. Conditions did not differ in sociodemographic or anthropometric variables, baseline food craving or hunger, or baseline HRV. However, clear differences in HRV were found between the six breaths and nine breaths condition during and after paced breathing, indicating that participants adhered to instructions, that is, adjusted their breathing rate according to the pacing bar and returned to their usual breathing rate afterwards.

State food craving dynamically changed throughout the experiment equally in both conditions. Food craving increased during paced breathing, when participants’ previously selected food picture was displayed on the screen, thus showing that the food pictures successfully induced craving. However, these changes in food craving were not influenced by breathing rate. Thus, slow breathing did not attenuate development of food craving during food-cue exposure, which is in line with speculations by May and colleagues (2010), where focusing on breath did not attenuate development of food craving in hungry individuals (but note that there was no instruction regarding breathing rate in that study).

Food craving decreased during both resting periods. While this was an unexpected finding, it is in line with studies showing that relaxation techniques in general may be beneficial for attenuating food craving and can positively influence eating behavior (Hamilton et al. 2013; Pawlow et al. 2003). At the end of the experiment, however, self-reported hunger remained elevated in the nine breaths condition, but not in the six breaths condition. Thus, slow breathing or HRV-BF may indeed have a beneficial effect for reducing current hunger after food-cue exposure. It needs to be stressed, however, that this effect was of small magnitude only (partial eta squared of smaller than 0.06; Cohen 1988), and, thus, replication is needed.

As mentioned above, no study to date has examined the immediate effects of slow breathing or HRV-BF on current food craving and, thus, literature on this topic is scarce. Therefore, the current study has more of a pilot character. Accordingly, numerous limitations have to be considered when interpreting current findings, but which also offer several avenues for future research. Firstly, only young women were investigated and, thus, results may differ in other samples, for example in men, or in clinical samples such as eating disorder or obese patients. Secondly, participants were not instructed to actively downregulate their craving, but only to adjust their breathing rate according to the pacing bar. The intention to regulate food craving may crucially change what effects slow paced breathing has on current food craving or hunger sensations. Thirdly, the food stimuli were presented throughout the entire paced breathing period. Accordingly, it is possible that ceiling effects have hampered an influence of slow breathing as strong food cravings were induced throughout. If craving is elicited by food-cues in daily life, it may be more beneficial to turn attention away from those stimuli and then use slowed breathing to reduce craving. This strategy may also be supported by results of the current study as craving decreased during both relaxation periods. Fourthly, we only compared two different breathing rates and results may be different with other breathing rates. Also, studies often determine individual breathing rates, which optimizes the effects of HRV-BF (i.e., increases in HRV; Lehrer et al. 2013). Fifthly, all participants were novices in slow paced breathing and, thus, results may be different in experienced subjects (Vaschillo et al. 2006). Finally, participants in the current study were food deprived for at least 1 h and mean food deprivation was about 5 h. While the exact physiological mechanisms of HRV-BF are not completely understood (Lehrer 2013), it gets even more complex when slow paced breathing is applied in the context of food and eating as the vagus nerve does, of course, not only innervate the heart, but also the gastrointestinal tract. Also, it has been found that a 24-h fast reduces HRV (Herbert et al. 2012). Thus, future studies may investigate food deprived versus sated individuals, which may reveal differential effects of slow paced breathing on current food craving or hunger.

To conclude, this study showed that state food craving and hunger dynamically changed across two relaxation periods and a paced breathing period during food-cue exposure. Breathing at six breaths per minute did not attenuate development of food craving during paced breathing, but appeared to have a delayed hunger-attenuating effect. Much more research is necessary to understand the mechanisms of slow paced breathing or HRV-BF and their effects on current food craving and hunger and we have suggested several lines of research that may be worth pursuing. Understanding these basic mechanisms may also be helpful for tailoring interventions based on slow paced breathing for the treatment of eating disorders or obesity (Meule et al. 2012a; Scolnick et al. 2014).

## Footnotes

1 Picture number in the *food.pics* database: 4, 6, 9, 10, 12, 26, 28, 32, 38, 41, 42, 60, 65, 68, 78, 82, 83, 85, 87, 89, 90, 101, 110, 111, 115, 129, 143, 150, 157, 158, 161, 167, 172, 173, 176, 293, 296, 309, 318, 465, 474, 556.

2 Note that results involving HRV data when using the incomplete data set with missing values were similar to results using the full data set with substituted values.

3 To examine a possible influence of trait food craving on state food craving development during the course of the experiment, we additionally calculated mixed effects models for each FCQ-S subscale separately. Models were fitted using the lme4 package for the R software (http://www.r-project.org). However, these analyses did not reveal significant interaction effects of time × FCQ-T-r scores or condition × time × FCQ-T-r scores (all *p*s > 0.05).

4 Another way to follow up this interaction would be to compare hunger scores after the second resting period (time 4) between conditions with ANCOVA using baseline hunger levels (time 1) as covariate. This revealed that participants in the nine breaths condition had higher hunger scores at the end of the experiment than those in the six breaths condition (*F*_(1,62)_ = 4.81, *p* = .03, η_p_² = 0.07).
